# Tuberous sclerosis complex: a case report and literature review

**DOI:** 10.1186/s13052-023-01490-z

**Published:** 2023-09-08

**Authors:** Yanlin Li, Zhihua Si, Wei Zhao, Cong Xie, Xu Zhang, Ju Liu, Jinzhi Liu, Zhangyong Xia

**Affiliations:** 1 Department of Neurology, Shandong Institute of Neuroimmunology, Shandong Key Laboratory of Rheumatic Disease and Translational Medicine, The First Affiliated Hospital of Shandong First Medical University & Shandong Provincial Qianfoshan Hospital, 16766 Jingshi Road, Jinan, 250014 China; 2 Department of Gerontology, Shandong Provincial Qianfoshan Hospital, The First Affiliated Hospital of Shandong First Medical University, 16766 Jingshi Road, Jinan, 250014 China; 3 Laboratory of Microvascular Medicine, Medical Research Center, Shandong Provincial Qianfoshan Hospital, Shandong University, 16766 Jingshi Road, Jinan, 250014 China; 4 Department of Neurology, Liaocheng People’s Hospital and Liaocheng Clinical School of Shandong First Medical University, 67 Dongchang West Road, Liaocheng, Liaocheng, 252000 China; 5 Department of Gerontology, Cheeloo College of Medicine, Shandong Provincial Qianfoshan Hospital, Shandong University, 44 Wenhua West Road, Jinan, 250012 China; 6 Department of Geriatric Neurology, Shandong Provincial Qianfoshan Hospital, The First Affiliated Hospital of Shandong First Medical University, 16766 Jingshi Road, Jinan, 250014 China; 7 Department of Neurology, Cheeloo College of Medicine, Liaocheng People’s Hospital, Shandong University, 44 Wenhua West Road, Jinan, 250012 China

**Keywords:** Tuberous sclerosis complex, Clinical manifestations, Neuroimaging manifestations, Misdiagnosis and overtreatment, Case report

## Abstract

Tuberous sclerosis complex (TSC) is an autosomal dominant disorder with different initial symptoms and complex clinical manifestations. A 14-year-old female patient presented with persistent fever and severe headache. Medical imaging examinations revealed multiple abnormal intracranial lesions. The patient had previously been misdiagnosed with “encephalitis and acute disseminated encephalomyelitis” after visiting numerous hospitals. Eventually, by combing the characteristics of the case and genetic testing results, the patient was diagnosed with TSC accompanied by Mycoplasma pneumoniae infection. The purpose of this case report and literature review is to improve understanding of the clinical diagnosis and treatment of TSC so as to avoid misdiagnosis, missed diagnosis, and overtreatment.

**Supplementary Information:**

The online version contains supplementary material available at 10.1186/s13052-023-01490-z.

## Background

Tuberous sclerosis complex (TSC) is an autosomal dominant disorder that is mainly characterized by mental retardation, intractable epilepsy, and facial angiofibroma [1]. The various clinical manifestations of TSC also include skin hypopigmentation, renal leiomyoma, and retinal hamartoma. Moreover, patients with TSC exhibit subependymal “candle-drip” shaped calcified nodules via brain computed tomography (CT) and multiple abnormal signals of long longitudinal relaxation times (T1) and transverse relaxation times (T2) via magnetic resonance imaging (MRI). This disease can be confirmed by gene diagnosis. Due to the variable initial symptoms of TSC, patients with TSC are initially diagnosed in different departments. As a result, misdiagnosis and underdiagnosis of this disease are frequent. This paper reports a case of TSC misdiagnosed as encephalitis and acute disseminated encephalomyelitis. It also reviews the diagnostic criteria and neuroimaging manifestations of TSC. The aim of this paper is to reduce the misdiagnosis and overtreatment of TSC by enhancing understanding of its typical characteristics.

## Case presentation

A 14-year-old female patient presented with persistent severe headache, no loss of consciousness, and no limb convulsions. She had developed a fever two weeks prior to admission, with a temperature that fluctuated between 37.8℃ and 39℃. The patient was initially diagnosed with encephalitis and acute disseminated encephalomyelitis due to multiple abnormal signals on her brain MRI. She received medication (specifics unclear) from a local hospital but showed no improvement. Thus, the patient was transferred to our hospital for further diagnosis and treatment.

The patient was a firstborn child, had a recent vaccination history, and had no history of hypoxia, family-hereditary disease, or infectious disease.

**Fig. 1:**
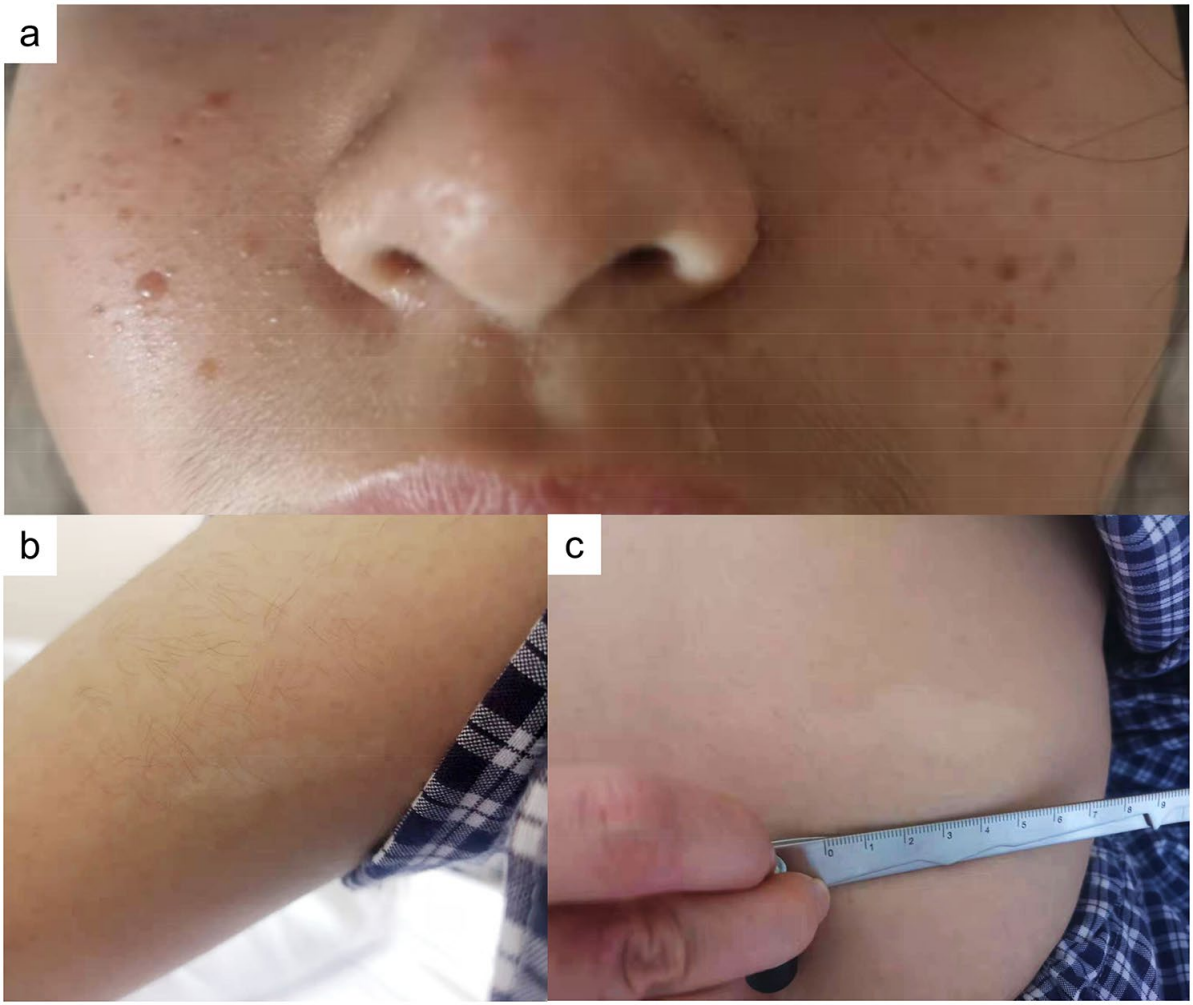
Skin involvement: **a** facial rashes, **b** hypopigmented macule on the left arm, and **c** hypopigmented macule on the back

The results of her physical examination following admission to our hospital are summarized in Table 1. The patient was conscious and fluent in the language, and no obvious abnormality was found during the physical examination of her advanced neurological function. A few slight pink rashes was observed on both her cheeks, and there were two hypopigmented macules (around 4 cm in length) on her left arm and back (Fig. 1). The patient’s Mini-Mental State Examination (MMSE) score was 28. The muscle strength and muscle tension of her limbs were normal, and the pathological signs and meningeal irritation signs were negative.

In terms of the patient’s auxiliary examination, blood biochemical indicator testing, C-reactive protein (CRP) analysis, erythrocyte sedimentation rate (ESR) assessment, TORCH [toxoplasma, others (such as syphilis and varicella zoster), rubella, cytomegalovirus, herpes simplex virus] infection screen, cerebrospinal fluid (CSF) analysis, CSF DNA assay for pathogens, CSF special protein analysis, CSF biochemical analysis, cryptococcal smear, general bacterial smear, fungal smear, acid-fast bacilli smear, cryptococcus neoformans capsular antigen determination, CSF high-throughput sequencing, and demyelinating antibody measurement were performed, and no obvious abnormalities were observed. The ratio of the patient’s Mycoplasma pneumoniae antibody was found to be > 1: 320 (< 1:40), which was positive, indicating the presence of Mycoplasma pneumoniae infection. The patient’s fever and headache symptoms were improved by treatment with 0.5 g of azithromycin per day.

**Fig. 2:**
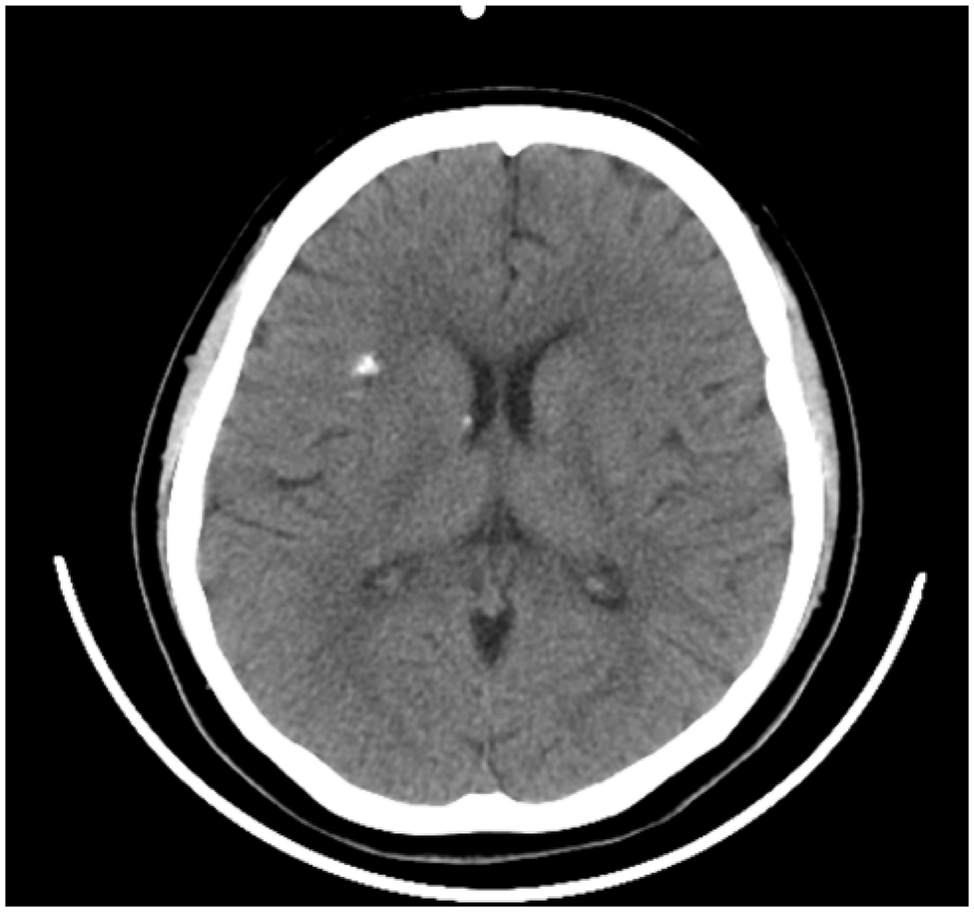
Subependymal “candle-drip” shaped calcified nodules in the right insula and ventricular wall

**Fig. 3:**
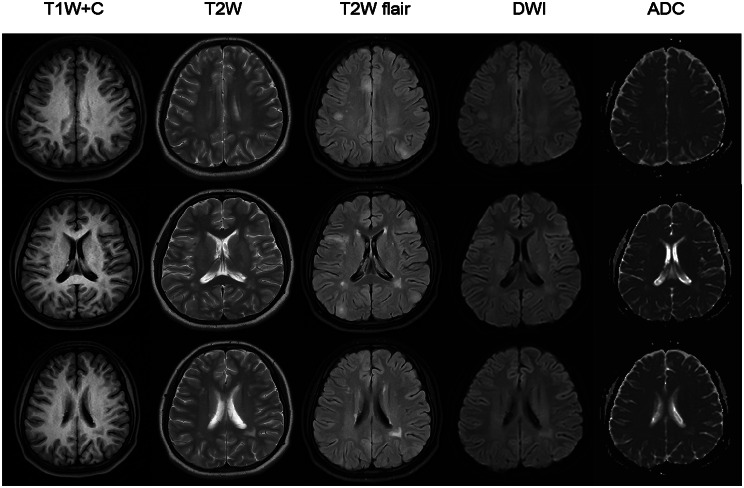
Multiple patchy and abnormal signals in the bilateral temporal lobe, hippocampus, bilateral frontal and parietal lobes, bilateral corona radiata, centrum semiovale region, and around the bilateral ventricles

Repeated craniocerebral CT showed punctate and patchy high-density shadows in the patient’s right insula lobe and ventricular wall (Fig. 2). In addition, MRI revealed multiple patchy and abnormal signals in the patient’s bilateral temporal lobe, hippocampus, bilateral frontal and parietal lobes, bilateral corona radiata, and centrum semiovale region, as well as around her bilateral ventricles (Fig. 3). Based on the characteristics of the case and the results of the imaging examinations, the analysis was performed and the patient’s diagnosis was determined according to the principle of midnights. First, with regard to central nervous system infection, the patient had a history of fever, a high mycoplasma infection index, and multiple intracranial abnormal signals. However, no obvious positive signs were found during her physical examination, while no obvious abnormalities were found in relation to her blood biochemical indicator level, CRP, ESR, procalcitonin, CSF DNA analysis results, CSF special protein, CSF biochemical analysis results, or high-throughput sequencing results and CSF demyelinating antibody. Hence, an infection of the central nervous system was not considered further. Second, in respect of central nervous system demyelinating diseases (e.g., acute disseminated encephalomyelitis and multiple sclerosis), the patient’s brain MRI showed multiple lesions, and she had a history of prodromal infection and recent vaccination. However, the whole onset of the disease was monophasic, while the patient’s peripheral white blood cell count, ESR, CSF pressure, and demyelinating antibody were all found to be normal, which did not support the diagnosis of a central nervous system demyelinating disease. Third, in terms of tumor-associated conditions, given that the patient had no obvious signs of focal localization and showed no mass effect or enhancement via MRI, such conditions were excluded. Fourth, concerning metabolic disorder or intoxication, there was no mention of any metabolic abnormalities or toxic exposure history in the patient’s medical history, and no metabolic abnormalities were found during the relevant examinations after her admission, which caused us to boldly discard such a possibility. In summary, after excluding the previously mentioned diagnoses, we considered the possibility of TSC.

**Table 1:** Patient characteristics

Temperature	Pulse	Respiration	Blood Pressure
37.5℃	96 cpm	18 cpm	138/82mmHg

**Fig. 4:**
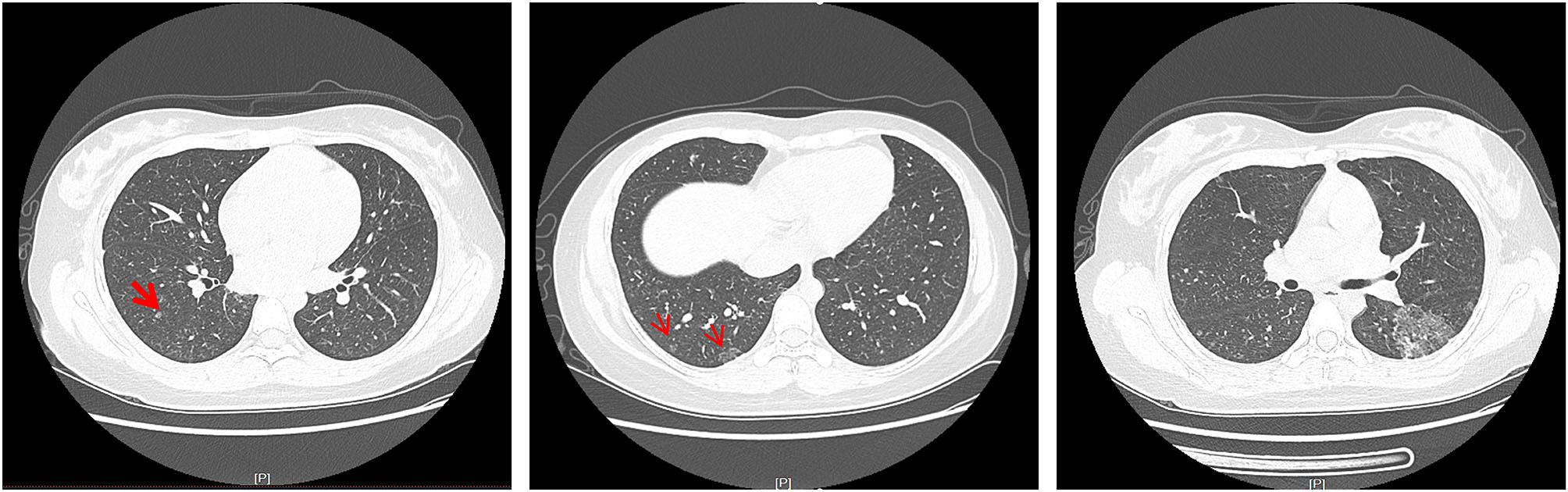
Chest CT examination: speckled, nodular, and flaky high-density shadows in the bilateral lungs

**Fig. 5:**
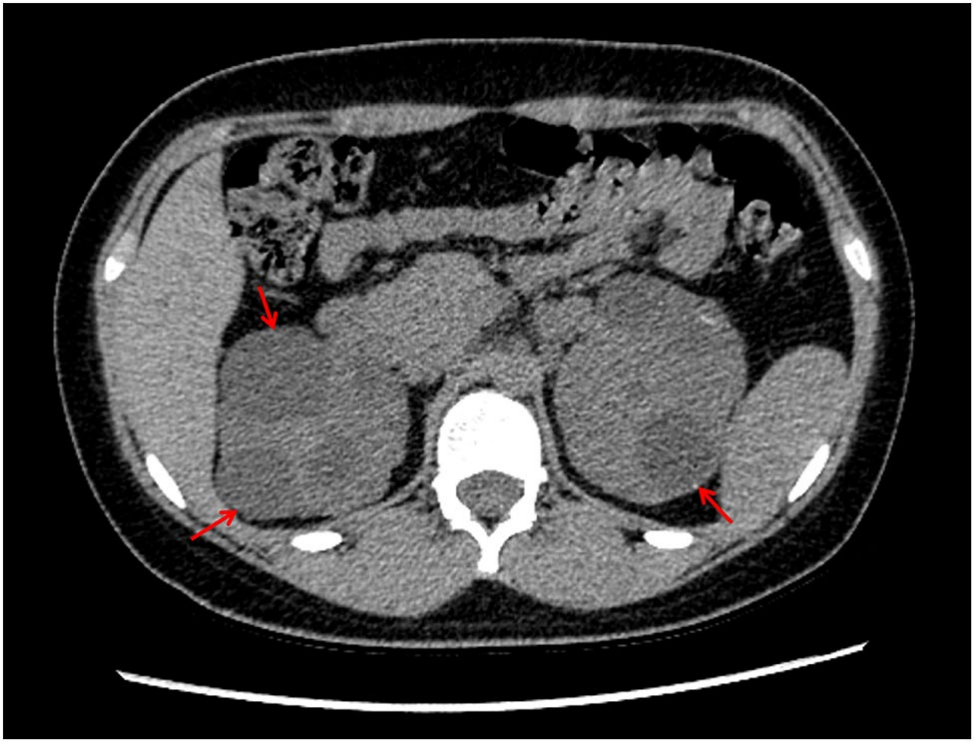
Abdomen CT examination: multiple cysts

After further improving the CT examination of the patient’s chest and abdomen, multiple speckled, nodular, and flaky high-density shadows were observed in her bilateral lungs (Fig. 4). Moreover, both her kidneys were enlarged in volume and irregular in appearance, and there were numerous cystic and water-density shadows of different sizes observed in the parenchyma of both kidneys (Fig. 5). However, no obvious abnormality was found during the fundus and electroencephalogram examinations. The detection of the whole exon gene suggested that there was a copy number variation with a deletion of a fragment size of 27 Kb in the chromatin 16p13.3 region, indicating a pathogenic mutation. Notably, the TSC2 gene related to TSC type 2 was localized in this region, which further confirmed the diagnosis of TSC. Based on these above examination results, the patient was given a final diagnosis of TSC with Mycoplasma pneumonia.

## Discussion

TSC is a congenital disorder caused by defects in the mTOR (mammalian target of rapamycin) pathway inhibitor TSC1/TSC2 complex. It has an incidence of 1/6000-1/10,000. There is no difference in the prevalence of TSC due to gender or race, although the symptoms are generally milder in women [2, 3]. Around 80% of patients are diagnosed in childhood, but diagnosis can be delayed until late childhood or adulthood when the typical neurological symptoms and skin features of the disorder disappear [4]. Early diagnosis can not only help to avoid unnecessary medical costs but also enhance the treatment and prognosis of TSC patients. To improve understanding of TSC, we reported the case of a 14-year-old female patient with persistent fever and severe headache who was initially misdiagnosed with encephalitis and acute disseminated encephalomyelitis upon admission.

Table 2 summarizes the criteria for the differential diagnosis of different diseases sharing an acute onset. Initial diagnosis of TSC is mainly based on clinical features. With the aid of medical imaging and genetics, it has been found that the symptoms of TSC extend far beyond the classic triad of facial angiofibroma, epilepsy, and mental retardation [5]. In fact, the common manifestations of TSC include cortical nodules, subependymal nodules (SENs), subependymal giant cell astrocytoma (SEGA), seizure, cardiac rhabdomyoma, renal angiomyolipoma, retinal hamartoma, pulmonary lymphangiomyoma, facial angiofibroma, intellectual disability, and autism spectrum disorder (ASD). In terms of its pathology, TSC is characterized by multiple benign tumors (hamartomas) and focal dysplasia lesions in various organs. The clinical manifestations of TSC are mostly associated with hamartomas, such as malformation, rupture, and pressure on surrounding normal tissues. Moreover, brain dysfunction (e.g., mental retardation, ASD) has no apparent causal relationship with anatomical damage. Therefore, the clinical manifestations of TSC can be classified into three categories: (1) hamartoma, (2) focal dysplasia, and (3) brain dysfunction [6].

**Table 2:** Differential diagnosis of different diseases sharing an acute onset

Disease	Clinical Symptoms	Imaging Features
Congenital TORCH	Infants infected with toxoplasma in utero often do not have obvious symptoms of toxoplasmosis at birth, and then they may gradually develop hepatosplenomegaly, jaundice, anemia, neurological disorders such as intracranial calcifications, hydrocephalus, and microcephaly, as well as complications such as chorioretinitis and learning disabilities. Maternal infection with rubella virus before 20 weeks of gestation can result in eye defects, congenital heart disease, sensorineural deafness, and central nervous system lesions in infants. The long-term sequelae may include diabetes, precocious puberty, and progressive panencephalitis. A small proportion of fetal infection with cytomegalovirus in utero can lead to growth restriction, microcephaly, intracranial calcification, hepatosplenomegaly, and long-term sequelae such as sensorineural deafness, visual impairment, psychomotor retardation, and learning disabilities.	Intracranial calcifications appear as linear calcified nodules, which are often accompanied by ventricular enlargement and patchy hypodense shadows in the periventricular white matter. Basal ganglia calcifications are also common and may be accompanied by brain atrophy and microcephaly.
Tuberous Sclerosis Complex	Skin manifestations include facial angiofibroma and hypopigmented macules on the trunk and limbs. Neurologic symptoms include epileptic seizures often accompanied by intellectual disability. The location of intracranial nodules can cause corresponding focal neurological deficits.	Calcified lesions are often located in the deep white matter of the hemisphere, frequently around the lateral ventricles, especially near the anterior and occipital horns and may be located in the centrum semiovale. Lesions are typically smaller in size (< 1.5 cm), with both new and old lesions being present, and have no surrounding edema or mass effect. They are typically arranged vertically against the ventricular lateral wall (Dawson finger sign) and are consistent with the course of the small blood vessels in the periventricular white matter. MRI is more sensitive than CT in detecting these lesions.
Cerebral Arteriovenous Malformation	Clinical symptoms commonly include cerebral hemorrhage, headache, and epileptic seizures.	Calcifications are typically ring or crescent-shaped, and they are accompanied by localized brain atrophy. Enhancement may be seen on contrast-enhanced scans.
Encephalofacial Angiomatosis (Sturge-Weber Syndrome)	Skin manifestations include flat facial hemangioma at birth, commonly located in the distribution area of the trigeminal nerve on one side of the face. Most of the hemangiomas distributed in the ophthalmic branch were purplish-red hemangiomas. In severe cases, the hemangiomas can spread over one side of the face. Neurologic symptoms include epileptic seizures, most of which are drug-resistant epilepsy. Mental decline often occurs with the increase of age, and there is a certain correlation between mental decline and the age of onset of the disease. The earlier the onset of the disease, the more likely to have mental decline. Hemiplegia and hemiatrophy may occur on the contralateral side of the cerebral facial hemangioma.	CT may show unilateral or localized brain atrophy, with the calcifications distributed in a gyriform pattern in the occipital and parietal cortex, often unilaterally. Hemangiomas may be present in the facial area ipsilateral to the lesion. MRI shows reticular enhancement of leptomeningeal vascular malformations. The ipsilateral choroid plexus is enlarged and significantly enhanced.
Cerebral Cysticercosis	The major manifestations include seizures, increased intracranial pressure, and meningoencephalitis. Patients may also experience tonic-clonic seizures, complex partial seizures, and other symptoms such as headache, vomiting, hemianopsia, and aphasia. Additionally, psychiatric disorders, paralysis, and cranial nerve palsy may occur.	Imaging findings vary with the stage of the disease and the host response. Typical presentation is in the subarachnoid space, and the cysticerci invasion can involve the cerebral cisterns, brain parenchyma, and ventricles. Intraventricular cysts are often solitary, with the fourth ventricle being the most common location for calcifications in individuals under 30 years old. Parenchymal cyst CT shows diffuse cerebral edema with well-defined gray-white matter boundaries, low-density lesions, and small cystic low density shadows, and narrowing of the sulcal fissures as well as the intraventricular system. The contrast-enhanced scans show no enhancement. Some patients may have pericystic edema with partial or complete calcifications. The CT results of the ventricular cysts show ovoid cystic low-density shadows in the enlarged ventricle, resembling cerebrospinal fluid, and small dot-like high-density shadows, which are cerebral cysticercus scoleces. Contrast-enhanced CT scans show no obvious enhancement. For the last type, that is, meningeal cyst, a hydrocephalus is present via CT, and enhanced CT shows varying degrees of meningeal enhancement reaction. The MRI manifestations of cerebral cysticercosis are diverse and can be classified into the survival stage and the death stage. The enhanced effect is obvious in the death stage, accompanied by edema. The enhancement is characterized by nodules of uniform size, with few exceeding 2.5 cm.
Neurofibromatosis Type 1	The characteristic feature is the presence of multiple non-malignant nervous system tumors. The two main manifestations are café-au-lait macules on the skin and multiple neurofibromas in the peripheral nerves. Patients may also exhibit symptoms such as epilepsy and learning disabilities.	Intracranial tumors may be associated with meningiomas and can be multifocal. They may also be combined with gliomas and ventricular meningiomas. CT findings shows non-neoplastic isolated calcifications in the temporal horn choroid plexus or calcifications along the entire choroid plexus. Hypoplasia of the greater wings of the sphenoid bone, combined with herniation of temporal lobe into the orbit and pulsatile proptosis, can be complicated by meningiomas, schwannomas, and gliomas. In terms of the MRI manifestations: (1) bilateral acoustic neuromas, most of which are more severe on one side; (2) multiple neurofibromas of the cranial and peripheral nerves; (3) concomitant meningiomas or gliomas; and (4) often accompanied by skeletal deformities, such as spinal bifida and skull defects.

Abbreviations: CT, computed tomography; MRI, magnetic resonance imaging

The revised diagnostic criteria for TSC published in 2012 (Table 3) include the following [5]: (1) pathogenic mutations in the TSC1 or TSC2 gene can confirm a diagnosis of TSC; (2) the presence of two main features, or the simultaneous occurrence of one main feature and two secondary features, proves the clinical diagnosis of TSC; and (3) the presence of one main feature or two secondary features can be suspected to indicate TSC [7, 8].

**Table 3:** Consensus statement of the 2012 International Tuberous Sclerosis Complex (TSC) Consensus Conference

Genetic Diagnosis	Clinical Diagnostic Criteria	
	Main Features	Secondary Features
There are definite loss-of-function mutations in the TSC1 and/or TSC2 genes with dysregulation of the mTOR pathway (which is sufficient but not necessary for the diagnosis of TSC). TSC1 and/or TSC2 mutations that are unclear or of no functional significance do not meet these criteria, and therefore, they cannot be used in the diagnosis of TSC.	Facial angiofibromas (≥ 3) or frontal fibrous plaque Hypopigmented macules (≥ 3 with a minimum diameter of 5 mm) Ungual fibromas (≥ 2) Shagreen patch or multiple collagenomas Multiple retinal nodular hamartomas Cortical dysplasia (cortical nodules or with white matter radial migration lines) Subependymal nodules Subependymal giant cell astrocytoma Cardiac rhambdomyoma Pulmonary lymphangioleiomyomatosis Renal angiomyolipomas (≥ 2)	Multiple dental enamel pits (≥ 3) Oral fibromas (≥ 2) Non-renal angioleiomyolipoma Multiple renal cysts Retinal achromic patch “Confetti” skin lesions

Abbreviation: mTOR, mammalian target of rapamycin

Following the development of both medical imaging and genetics, advanced imaging techniques now contribute to the diagnosis of TSC, with neuroimaging in particular being essential for its early diagnosis, monitoring, and treatment. In 2016, Konakondla et al. reported a case of TSC with isolated subependymal giant cell astrocytoma as the first imaging manifestation, which further demonstrated the critical role of imaging in the identification of this disease [9]. There are four main neuroimaging manifestations of TSC namely, cortical tubers, white matter lesions (WMLs), SENs, and SEGA [10–14]. Cortical tubers are glial brain hamartomas, which can implicate both gray and white matter, and their production may be associated with the overexpression of microRNA-34a in cortical cells [15]. The distribution of cortical tubers is generally limited to the frontal and parietal lobes, although it can also involve the entire brain. According to MRI findings, cortical tubers can be divided into three types: (1) type A cortical tubers have iso-signal intensity on T1, high-signal intensity on T2/FLAIR, and no increased diffusion on ADC images; (2) type B cortical tubers have low-signal intensity on T1, high-signal intensity on T2/fluid attenuated inversion recovery (FLAIR), and no enhanced diffusion on ADC images; and (3) type C cortical tubers have hypointensity on T1, hyperintensity with hypointense cores and inhomogeneous halos on T2/FLAIR, and enhanced diffusion on ADC images [13, 14]. Clinicians who are inexperienced in relation to the neuroimaging features of TSC are prone to ignore the imaging manifestations of type A cortical tubers and draw the incorrect conclusion that MRI results are “negative” during clinical diagnosis and treatment.

In addition to the common neuroimaging features, approximately 1%–5% of TSC patients exhibit rare neuroimaging manifestations, including parenchymal calcification, hemiencephaly, mild dilatation of the lateral ventricles caused by atrophy or dysplasia, Chiari malformation, microcephaly, macrocephaly, arachnoid cysts, neurofibroma, and chordoma [10–14, 16]. The following rare neuroimaging findings are closely associated with TSC.

### Brain aneurysm

Angiomyolipoma associated with TSC is prone to form microhemangioma due to the poor elasticity of the vascular wall, resulting in hemangioma rupture and bleeding. In TSC patients’ medical records, aortic aneurysm, and intracranial aneurysm represent the main cases, while central aneurysm, peripheral aneurysm, and steno-occlusive arteriopathy of the large and medium-sized arteries are occasionally reported. Most patients with brain aneurysm are young children and women. The incidence of brain aneurysm increases with age, and it often manifests as fusiform aneurysms, giant aneurysms, or multiple aneurysms [17, 18].

### Arachnoid cyst

In TSC patients, men and those with continuous deletions of the TSC2-PKD1 gene are more likely to develop an arachnoid cyst, which may be related to neural crest dysplasia. In addition, the arachnoid cyst may not be concomitant with cortical tubers, SENs, and WMLs, and it usually occurs on the side of the face with severe skin lesions [18, 19].

### Enhanced cystic lesions of the white matter

White matter cystic lesions are cysts characterized by a clear periventricular boundary, iso-signal intensity of CSF, and no contrast enhancement. They are round or ovoid in shape and around 2-12 mm in size, including cortical tuber-associated WMLs, radial white matter bands, and cystic WMLs [12, 20]. In 2021, D’Amico et al. reported two cases of enhanced cyst-like lesions of the white matter after the injection of the gadolinium contrast agent. After excluding cerebral infarction, Virchow Robin space dilatation, hair cell astrocytoma, and high-grade glioma, enhanced cystic lesions of the white matter were considered as a novel neuroimaging manifestation of TSC [21]. However, the specific mechanism of enhanced cystic lesions of the white matter is not clear, although it may be related to an increase in blood-brain barrier permeability.

TSC often involves diverse organs, and the treatment of newly diagnosed cases of TSC is usually multidisciplinary, which poses a challenge for the comprehensive clinical management of TSC patients. Traditionally, TSC therapy mainly consists of surgical treatment and symptomatic supportive treatment. However, in recent years, the concept of precision medicine has been proposed, and increasing attention has been paid to molecular targeted therapy. Given that the pathogenesis of TSC is related to the loss of the negative regulatory function of the TSC1/TSC2 complex on the mTOR pathway, mTOR inhibitors are considered ideal targeted drugs for the treatment of TSC. They are also are widely used to treat TSC-related refractory epilepsy, facial fibroangioma, renal angiomyolipoma, and other diseases [22]. Due to the deepening of research in this area, more targeted therapies have emerged. For example, diacylglycerol kinase alpha (DGKA) inhibitors can be applied for the targeted treatment of TSC via macropinocytosis of TSC2-deficient cells [23], while microRNA-34a inhibitors can be utilized for early intervention in relation to TSC-related neurological symptoms [15] and treatment for TSC-related renal angiomyolipoma by regulating the lipid metabolism of fibroblasts [24]. Recent studies have found that heat shock protein-90 (HSP90) inhibitors have suppressive effects on TSC1/TSC2-deficient cell lines, while HSP90 dysregulation may be involved in the pathogenesis of TSC-associated renal angiomyolipoma. Thus, HSP70 has become a potential target for the treatment of TSC-related hamartoma [25, 26].

In the reported case, the patient had symmetrical, reddish, hard, and waxy papules that protruded from the skin surface and ranged in size from needle-like to broad-bean-like, on both cheeks. Of note, there were two hypopigmented macules (around 4 cm in length) on the left arm and back of the patient. In addition, the patient’s cranial CT showed insula and subependymal calcifications, while her cranial MRI revealed cortical tubers, SENs, and WMLs. Furthermore, we observed multifocal micronodular pulmonary histiocytosis and multiple renal cysts via ranal CT. Therefore, the reported case met the clinical diagnostic criteria set out in the Consensus Statement of the 2012 International TSC Consensus Conference. As a consequence, the possibility of TSC was raised (Table 3). The whole-exome genetic testing results suggested that the patient had a copy number variation with a deletion of a fragment size of approximately 27 Kb in the 16p13.3 region, indicating a pathogenic mutation. Notably, the TSC2 gene associated with TSC type 2 was localized in this region, which further confirmed the diagnosis of TSC. It is worth mentioning that around 80% of SENs are calcified tumors, which may progress into SEGA and block the foramen of Monro, leading to obstructive hydrocephalus [7]. In addition, the continuous deletion of the TSC2 and PKD1 genes increases the risk of an arachnoid cyst developing. Hence, regular brain MRI is necessary for this patient.

The limitation of this case report lies in the fact that it did not consider whether the patients exhibited growth retardation. Given that the patient had no significant difference from other children of the same age in terms of growth and intelligence and that her MMSE score was 28, her head circumference was not measured. In future clinical work, we will pay more attention to children’s growth and development detection. Moreover, additional attention will be paid to the detection of all TSC patients’ growth and development.

## Conclusion

We describe a case of TSC initially misdiagnosed as encephalitis and acute disseminated encephalomyelitis, which was finally diagnosed via reexamination of the patient’s brain CT and MRI results combined with genetic detection results. TSC has a low incidence and diverse phenotypes. Misdiagnosis can easily occur in patients with fever and scattered abnormal signals on cranial MRI who present without the typical clinical features and/or family history. Consequently, the possibility of TSC should be considered in relation to patients with a history of both infection and vaccination, multiple scattered papules on the face, and multiple abnormal signals on MRI.

### Electronic supplementary material

Below is the link to the electronic supplementary material.

Supplementary Material 1

## Data Availability

The data that support the findings of this study are available from the corresponding author upon reasonable request. *Competing interests*. The authors declare that there are no conflicts of interest.

## Declarations

*Ethics approval and consent to participate*.

Our study was approved by the Ethics Committee of The First Affiliated Hospital of Shandong First Medical University & Shandong Provincial Qianfoshan Hospital. And the written informed consent to participate was provided by all participants. All procedures performed in this study were in accordance with the ethical standards of the institutional and/or national research committee and with the 1964 Helsinki declaration and its later amendments or comparable ethical standards.

*Consent for publication*.

The written informed consent for publication was obtained from all participants.

## List of abbreviations

TSC: Tuberous sclerosis complex
MRI: magnetic resonance imaging
CT: computed tomography
CRP: C-reactive protein
ESR: erythrocyte sedimentation rate
CSF: cerebrospinal fluid
WMLs: white matter lesions
SENs: subependymal nodules
mTOR: mammalian target of rapamycin
SEGA: subependymal giant cell astrocytoma
ASD: autism spectrum disorder
FLAIR: fluid attenuated inversion recovery
DGKA: diacylglycerol kinase alpha
HSP90: heat shock protein-90
