# Supplementary material for: Tuberous sclerosis complex: a case report and literature review
Source: Ital J Pediatr. 2023 Sep 8;49:116. doi: 10.1186/s13052-023-01490-z (PMC10485941; doi:10.1186/s13052-023-01490-z)
Supplement: Supplementary file 1 — Supplementary Material 1 [file 13052_2023_1490_MOESM1_ESM.docx]

Dear editors and reviewers,

Thank you very much for your kind reply to our manuscript. The authors greatly appreciate the reviewers’ careful reading of the manuscript and their constructive suggestions. According to your advice, we have tried to revise the manuscript. The point-by-point responses to your comments are as follows and the changes are made and marked in the revised manuscript.

Responses to reviewers’ concerns are in Red. Reviews in their entirety are in non-red type.

Reviewer reports:

Reviewer #1: the manuscript could be improved in the discussione section of a table summering the differential diagnosis with tSC sharing the acute onset.

**Response:** Thanks for your valuable advice. Please see Table 2 that summarizes the differential diagnosis with different diseases sharing the acute onset. The diagnosis of TSC is discussed in detail from the second half of page 6 to page 8.

We appreciate the editors’ and reviewers’ warm work earnestly and hope that the correction will meet with approval.

Once again, thank you very much for your comments and suggestions.

Sincerely yours,

Jinzhi Liu and Zhangyong Xia
